# Brain state-dependent abnormal LFP activity in the auditory cortex of a schizophrenia mouse model

**DOI:** 10.3389/fnins.2014.00168

**Published:** 2014-07-01

**Authors:** Kazuhito Nakao, Kazu Nakazawa

**Affiliations:** ^1^Department of Psychiatry and Behavioral Neurobiology, University of Alabama at BirminghamBirmingham, AL, USA; ^2^Unit on Genetics of Cognition and Behavior, Department of Health and Human Services, National Institute of Mental Health, National Institutes of HealthBethesda, MD, USA

**Keywords:** auditory steady-state responses, GABAergic interneurons, gamma oscillation, local field potentials, NMDA receptors, parvalbumin, schizophrenia, mouse models

## Abstract

In schizophrenia, evoked 40-Hz auditory steady-state responses (ASSRs) are impaired, which reflects the sensory deficits in this disorder, and baseline spontaneous oscillatory activity also appears to be abnormal. It has been debated whether the evoked ASSR impairments are due to the possible increase in baseline power. GABAergic interneuron-specific NMDA receptor (NMDAR) hypofunction mutant mice mimic some behavioral and pathophysiological aspects of schizophrenia. To determine the presence and extent of sensory deficits in these mutant mice, we recorded spontaneous local field potential (LFP) activity and its click-train evoked ASSRs from primary auditory cortex of awake, head-restrained mice. Baseline spontaneous LFP power in the pre-stimulus period before application of the first click trains was augmented at a wide range of frequencies. However, when repetitive ASSR stimuli were presented every 20 s, averaged spontaneous LFP power amplitudes during the inter-ASSR stimulus intervals in the mutant mice became indistinguishable from the levels of control mice. Nonetheless, the evoked 40-Hz ASSR power and their phase locking to click trains were robustly impaired in the mutants, although the evoked 20-Hz ASSRs were also somewhat diminished. These results suggested that NMDAR hypofunction in cortical GABAergic neurons confers two brain state-dependent LFP abnormalities in the auditory cortex; (1) a broadband increase in spontaneous LFP power in the absence of external inputs, and (2) a robust deficit in the evoked ASSR power and its phase-locking despite of normal baseline LFP power magnitude during the repetitive auditory stimuli. The “paradoxically” high spontaneous LFP activity of the primary auditory cortex in the absence of external stimuli may possibly contribute to the emergence of schizophrenia-related aberrant auditory perception.

## Introduction

Neural oscillation and synchronization abnormalities have been suggested to play a role in the information and sensory processing deficits commonly seen in schizophrenia (Ford and Mathalon, 2008; Uhlhaas and Singer, 2010; Gandal et al., 2012a). Periodic auditory stimulation entrains the electro-encephalogram (EEG) to a specific phase and frequency, often referred to as the auditory steady-state response (ASSR). In both human and animal models, the ASSR has been used to assess the functional integrity of neural circuits that support synchronization (Picton et al., 2003; Brenner et al., 2009; O'Donnell et al., 2013). In schizophrenia, reduced ASSR power (magnitude) and phase locking (phase consistency across trials), particularly at 40 Hz, are observed in EEG (Kwon et al., 1999; Brenner et al., 2003; Light et al., 2006; Spencer et al., 2008, 2009; Vierling-Claassen et al., 2008; Krishnan et al., 2009) as well as in magneto-encephalogram (MEG) (Teale et al., 2008; Maharajh et al., 2010; Tsuchimoto et al., 2011) studies. Since cortical parvalbumin (PV)-positive fast-spiking interneurons have an intrinsic resonance near this range (Tateno et al., 2004; Golomb et al., 2007), the reduction in 40-Hz ASSRs may reflect functional deficits of these fast-spiking neurons in schizophrenia.

Earlier studies of gamma synchrony deficits in schizophrenia reported the *relative* changes in gamma band activity in response to task stimuli, by assessing stimulus-evoked responses in synchrony compared with a pre-stimulus baseline (Kwon et al., 1999; Haig et al., 2000; Lee et al., 2003). Thus, in these studies *relatively* less evoked gamma synchrony could be a reflection of greater baseline spontaneous gamma phase synchrony under pre-stimulus conditions. However, in schizophrenia the evidence regarding baseline gamma activity abnormalities is inconsistent. Both increases (Jalili et al., 2007; Venables et al., 2009; Kikuchi et al., 2011; Spencer, 2012) and decreases (Yeragani et al., 2006; Rutter et al., 2009) in *baseline* spontaneous gamma power during pre-stimulus period or “resting state” have been reported. The reason for these contradictory results has yet to be clarified.

To measure the baseline spontaneous gamma band power with high precision, it would be useful to directly record local field potentials (LFPs), necessitating the use of animal models. To that end, we recorded LFPs directly from the primary auditory (A1) cortex of GABAergic interneuron-specific NMDA receptor (NMDAR) hypofunction mice (*Ppp1r2*-cre/fGluN1 KO mice). Previous studies using this mutant mouse revealed that the selective deletion of GluN1, an indispensable subunit of NMDARs, in cortical and hippocampal interneurons during early postnatal development recapitulates several schizophrenia-like behavioral and pathophysiological phenotypes (Belforte et al., 2010; Jiang et al., 2013). In the present study, we subjected these mutant mice to the ASSR paradigm, similar to the one used in human studies (Krishnan et al., 2009). We assessed the auditory click train-evoked ASSRs and baseline LFP fluctuations in pre/post-stimulus period and at baseline (i.e., between stimulus presentations).

## Materials and methods

All experimental procedures were in accordance with National Research Council guidelines for the care and use of laboratory animals, and were approved by the National Institute of Mental Health Animal Care and Use Committee. Data analysis was conducted at the University of Alabama at Birmingham.

### Animal

*Ppp1r2*-cre^(+/−)^/fGluN1^(f/f)^ mice (henceforth referred to as KO mice or mutants) were generated as previously described (Belforte et al., 2010). Briefly, the protein phosphatase 1, regulatory subunit 2 (*Ppp1r2*)-cre line and a floxed-GluN1 (fGluN1) line were used to delete exons 9 and 10 of GluN1 gene from the postnatal second week in a subset of cortical and hippocampal Ppp1r2-cre positive interneurons, the majority of which are PV-positive. Female mutant mice were bred to homozygously fGluN1 male mice to generate the same mutant and fGluN1 control mice with a 50% probability. In the present study, 65 male mice received chronic survival surgery for the microwire array implantation. After successful detection of the auditory-evoked potentials 1 week after the surgery, 7 fGluN1 control (13–16 week-old, 30.6 ± 0.65 g body weight) and 6 mutant (12–14 week-old, 28.1 ± 0.8 g) mice were subjected to in-depth analysis of ASSRs, as described in Result section.

### Surgical procedures

Animals were anesthetized with isoflurane to surgical levels and were mounted in a stereotaxic instrument with non-rupture ear bars (Zygoma ear cups, David Kopf Instruments). A custom-made plastic headpost was secured to the occipital bone at the midline with superglue and dental acrylic, and was used to fix the animal's skull to the stereotaxic instrument. This was done to prevent physical occlusion of the external ear canals by stereotaxic ear bars in order to obtain tone-evoked LFP responses. A unilateral craniotomy was made over the right temporal bone from 1.5 to 3.5 mm posterior to bregma and from 3.5 to 4.5 mm lateral to midline. The vasculature was inspected. The microwire multi-electrode array consisted of six tetrodes, which were custom-configured in a 2 × 3 matrix with inter-electrode distance of ~200 μm, covering 0.6 × 0.8 mm^2^. The impedance of each electrode was between 0.2 and 0.3 MΩ. The microwire array was inserted into the superficial layers of A1 cortex with the aid of cortical vascular patterns, and two stainless steel screws in the frontal cortex which served as ground and reference electrodes. After the dosage of isoflurane was reduced to 1%, a single white noise pulse (1 ms, duration; 80 dB, SPL) was applied to activate the A1 cortical area. In order to allow for the tone-evoke responses it is critical to maintain the isoflurane concentration at 1% (Santarelli et al., 2003). The animal was held in place with adhesive tape to prevent head twitching or grooming. An analgesia (buprenorphine, 0.1 mg/kg *s.c*.) was given to diminish pain sensation during the surgery. If single-tone evoked potentials (over 0.1 mV magnitude) were detected in at least one electrode of the microwire array, the electrodes were inserted further until maximal responses were obtained. The anesthetic dose was then returned back to surgical levels, and the microwire array was fixed to the skull with dental acrylic.

### *In vivo* recording

Seven days after surgery, LFP recording was performed from A1 cortex of awake, head-restrained mice. The mice were briefly anesthetized with 1% isoflurane to hold the animal head fixed to the stereotaxic instrument using the headpost, and the body was covered with adhesive paper tape to limit body movements. The micro-array electrodes were directly connected, via an EIB-27-Micro headstage pre-amplifier, to a Cheetah-64 recording system (Neuralynx Inc.), where LFP signals were filtered (bandwidth from 0.1 to 475 Hz), digitized, and acquired at a sampling rate of 1.56 kHz per channel. Thirty minutes after the cessation of anesthesia, LFP recording began from A1 cortex of awake, head-restrained mice in a custom-made auditory isolation chamber (background sound level, 40 dB SPL).

In the first session, spontaneous LFP activity during a pre-stimulus period was recorded from A1 cortex for 2–25 min. Subsequently, in the second session, 500-ms long click trains consisting of 80 dB white-noise pulses presented at 40 Hz (40-Hz ASSR stimuli) were applied 50 times with an inter-stimulus interval of 20 s, which mimics the ASSR protocol used in human studies (Krishnan et al., 2009). Auditory click stimuli, consisting of white noise pulses (1 ms, duration; 80 dB, SPL), were generated in Labview (National Instruments Inc.), and presented using a speaker with a 35 Hz–20 kHz frequency response (Z3, Logitech Inc.) placed 30 cm above the mouse head. In the third session, which began 10 min after cessation of the second session, 1000-ms long click trains consisting of 80 dB white-noise presented at 20 Hz (20-Hz ASSR stimuli) were applied 50 times with an inter-stimulus interval of 20 s. In the last session, spontaneous LFP activity was recorded for 25 min as a post-stimulus period. When no auditory evoked LFP responses were detected in any channels during the second session, the experiment was terminated and the animal was euthanized.

### LFP analysis

Only the channel data in which the amplitude of initial N1 response in the 40 Hz-ASSRs was more than 0.1 mV (~4 times the standard deviation), were used for subsequent analyses. Neuralynx LFP files were first converted to Spike2 format to visually inspect the raw data. Next, LFP voltage values in the Neuralynx files were converted to Matlab (Mathworks) files, and these values were normalized to the z-scores by subtracting the mean and dividing by the standard deviation of the LFP voltages during entire recording epoch (~20 min). The Matlab files with the z-scores were then converted to NeuroExplorer (Nex Technologies) files to calculate the power.

**Figure 1:**
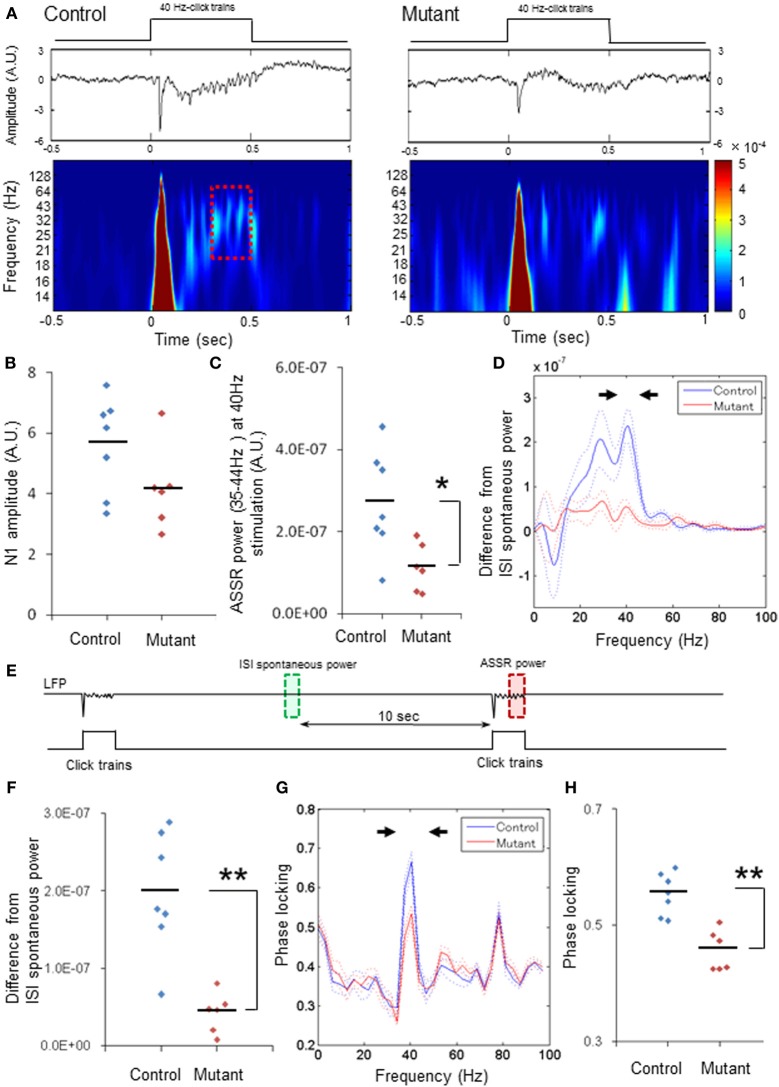
**Robust reduction in power and phase-locking of 40-Hz ASSRs. (A)** Representative examples of the averaged 40-Hz ASSR (middle, z-score) and spectrogram (bottom) in response to 40-Hz click trains (upper; 80 dB intensity, 500 ms duration). Time 0 is tone onset. **(B)** No difference in the averaged N1 amplitudes (z-score) evoked by 40-Hz click trains between genotypes (blue for 7 fGluN1 control mice; red for 6 mutant mice). *p* = 0.11, unpaired Student's *t*-test **(C)** Evoked ASSR power (z-score) at 35–44 Hz frequency range during 40-Hz click train stimulation in mutants (red) was lower than controls (blue). ^*^p < 0.05, unpaired Student's *t*-test. **(D)** The mean difference (A.U.) from baseline spontaneous power during inter-stimulus interval (ISIs; green square in Panel **E**) in click train-evoked ASSR power during last 200 ms before cessation of 40-Hz click trains (red square in Panels **A,E**). Dotted lines: mean ± s.e.m. **(E)** Schematic diagram indicates the analysis periods of baseline LFP power (green, ISI spontaneous power) and the evoked ASSR power (red). Relative ASSR power amplitudes shown in Panel **(D)** were calculated by subtracting an ISI power (in green) from the evoked ASSR power (in red) for each channel, and averaged per animal. **(F)** The difference in the magnitude between 35 and 44 Hz spectral power (arrowheads in Panel **D**) and the baseline for 40-Hz ASSRs in mutants (red) was lower than controls (blue). ^**^p < 0.01, unpaired Student's *t*-test. **(G)** Phase locking to 40-Hz steady-state tone stimuli in control (blue) and mutant (red) mice. Dotted lines: mean ± s.e.m. **(H)** Magnitude of 35–44 Hz phase locking for 40-Hz ASSRs (arrowheads in Panel **G**) in mutants (red) was lower than controls (blue). ^**^p < 0.01, unpaired Student's *t*-test. Each dot represents individual animals. Dotted lines in Panels **(D,G)** are s.e.m.

In order to assess the oscillatory component of evoked ASSRs, z-score normalized LFPs during the last 200-ms of each ASSR were analyzed with a fast Fourier transform (FFT) algorithm in the range of 0–100 Hz using 256 frequency bins and presented as total ASSR power (e.g., Figure 1C). Relative power amplitudes were calculated by subtracting a baseline spontaneous power, which was from the 200-ms inter-stimulus segment 10 s prior to each click-train onset, from the total ASSR power (see Figure 1E).

For spontaneous LFP power during a pre-stimulus period, LFP data (200-ms bin) during the last 10 s prior to the first click-train administration were analyzed with FFT algorithm in the range of 0–200 Hz using 256 frequency bins (**Figure 3**). To compare the baseline power magnitudes in-between ASSR sessions with the spontaneous power during the pre- or post-stimulus period, z-score normalized LFP from 5 to 15 s (200-ms bin) after 1st stimuli, 25th stimuli, and 50th stimuli were analyzed with FFT algorithm in the range of 0–100 Hz using 256 frequency bins. For the pre-stimulus period, the baseline spontaneous power during last 10 s before click train onset was analyzed in the range of 0–100 Hz using 256 frequency bins. For power spectral analysis during the post-stimulus period, LFP data obtained from a 10-s period (200-ms bin) 20 min after the cessation of all ASSR stimuli were analyzed with FFT algorithm in the range of 0–100 Hz using 256 frequency bins (see **Figure 4A**).

To calculate phase locking to auditory click- trains, phase locking was performed in a frequency range 0–100 Hz with a 60% overlapping window after applying Hanning tapering of normalized LFP data, which was further analyzed with FFT algorithm. To plot a scalogram (wavelet spectrogram), Matlab z-score files of LFP were wavelet transformed using a Complex Gaussian wavelet from Matlab wavelet toolbox.

### Statistics

Given that between-animal variability may be larger than within-animal variability in per-channel (i.e., per electrode) design, we mainly presented the data with per-animal design in the Figures and some data with per-channel design in the Supplemental Figures (see Lazic and Essioux, 2013). Differences between groups were assessed for normally distributed data using a Student's *t*-test (Statcel 2nd ed., OMS, Tokyo, Japan). The effect size was assessed as Cohen's *d*. For the graph data in **Figures 4**, **5**, differences were assessed by repeated measures of ANOVAs followed by Bonferroni *post-hoc* analysis (SPSS, IBM). Data were presented as mean ± s.e.m.

## Results

### Robust reduction of 40-HZ auditory steady-state responses (ASSRs)

Seven days after surgery, 40-Hz click train-evoked initial N1 responses (i.e., the transient auditory evoked potentials to click train onset, more than 0.1 mV) were detected in A1 cortex from13 mice (7 fGluN1 control and 6 mutant mice), out of a total of 65 animals in which the click-train-evoked responses had been detected during the electrode implantation surgery. The relative high number of animals that displayed no evoked LFPs was mostly likely to be due to a shift of or damage to the electrode microarray placed on the temporal bone. Thirty-one LFP recordings from 7 control mice [animal #1: 2 (number of recording sites to be analyzed)/6 (total channel number), #2: 6/6, #3: 3/6, #4: 6/6, #5: 6/6, #6: 3/6, #7: 5/6], and 26 LFP recordings from 6 mutant mice (animal #1: 5/6, #2: 4/6, #3: 6/6, #4: 5/6, #5: 3/6, #6: 3/6) were subjected to subsequent LFP in-depth analysis.

Fifty 40-Hz click trains (duration, 500 ms) were delivered to click train-naïve animals with an inter-stimulus interval of 20 s. Figure 1A depicts representative examples of the averaged ASSRs (middle) and scalogram (wavelet spectrogram, bottom) evoked by 40 Hz stimulation (upper) in the floxed-control (left) and mutant mouse (right). Robust click train-evoked N1 potentials were elicited within the first 100 ms after click-train onset in both genotypes, and there were no differences in the averaged N1 amplitudes between genotypes per animal (Figure 1B). However, the N1 amplitudes averaged per channel were lower in the mutants compared to the floxed-control mice (*p* < 0.05, Student's *t*-test, Supplemental Figure 1A). To assess the subsequent ASSRs coherent to the 40-Hz click trains without any impact of evoked N1 potentials on the steady-state responses, LFP data (z-score) during last 200 ms before click-train cessation (a dashed line period in Figure 1A) were analyzed with an FFT algorithm. We found that the amplitudes of 40-Hz ASSRs were smaller in the mutants compared to the controls per animal [Figure 1C, *t*_(11)_ = 2.8, *p* < 0.05, Cohen's *d* = 1.60 (large effect size)] and per channel [Supplemental Figure 1B, *t*_(55)_ = 5.23, *p* < 0.01, *d* = 1.43 (large effect size)]. Difference in evoked ASSR power from baseline spontaneous power during inter-stimulus intervals, which were obtained by subtracting the spontaneous power amplitudes in-between ASSR stimuli from total ASSR power (Figure 1E), also peaked at 40 Hz and, to the lessor degree, at 30 Hz in the controls. Conversely, only small differences were detected in the mutant mice (Figure 1D and Supplemental Figure 1C). Figure 1F and Supplemental Figure 1D showed power spectrum density difference from the baseline at 35–44 Hz for each animal [*n* = 7 controls, *n* = 6 mutants, *t*_(11)_ = 4.57, *p* < 0.01, *d* = 2.64 (large effect size)] and for each channel [*n* = 31 sites from 7 controls, *n* = 26 sites from 6 mutants, *t*_(55)_ = 7.79, *p* < 0.01, *d* = 2.14 (large effect size)], indicating that average low gamma power for the evoked ASSRs was lower in the mutants compared to the controls. In addition, phase locking analysis of the 40-Hz ASSRs (z-score) revealed two peaks at 40 Hz (35–44 Hz) and at 80 Hz (75–84 Hz) for both controls and mutants (Figure 1G), but only phase locking at 40 Hz in the mutants was lower in comparison to controls for each animal [Figure 1H, *t*_(11)_ = 4.93, *p* < 0.01, *d* = 2.75 (large effect size)] and for each channel [Supplement Figure 1E, *t*_(55)_ = 9.42, *p* < 0.01, *d* = 2.47 (large effect size)]. These findings suggest that mutants are severely impaired in 40-Hz ASSR for both amplitude and phase locking, both of which are reminiscent of ASSR deficits in schizophrenia patients.

### Diminished 20-HZ ASSR power and phase-locking

**Figure 2:**
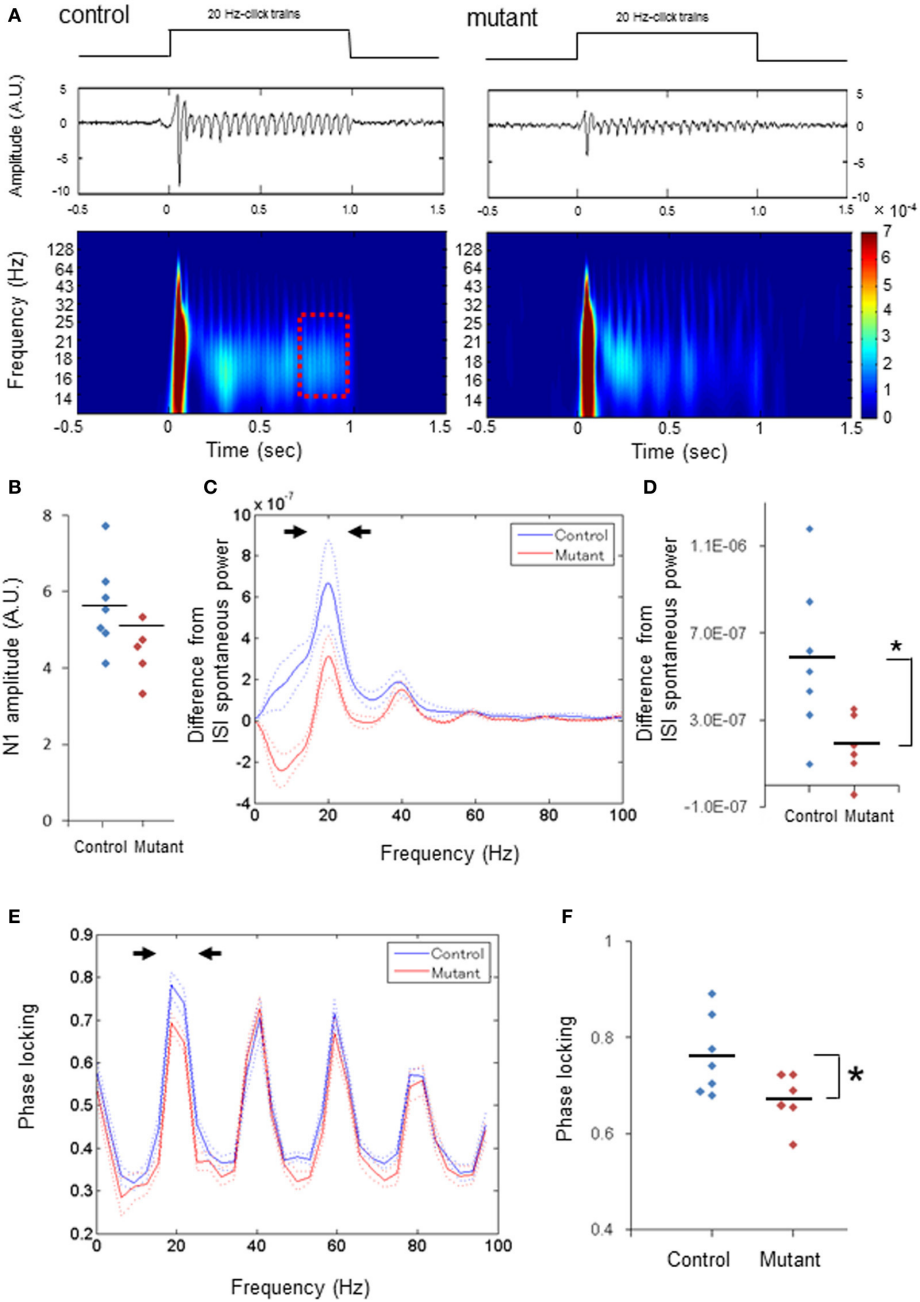
**Diminished power and phase-locking of 20-Hz ASSRs. (A)** Representative examples of the averaged 20-Hz ASSR (middle, z-score) and spectrogram (bottom), in response to 20-Hz click trains (upper; 80 dB intensity, 1000 ms duration). Time 0 is tone onset. **(B)** No difference in the averaged N1 amplitudes (z-score) evoked by 20-Hz click trains between genotypes (blue for 7 fGluN1 control mice; red for 6 mutant mice). *p* = 0.54, unpaired Student's *t*-test. **(C)** The mean difference (A.U.) from ISI spontaneous power in click train-evoked ASSR power during last 200 ms before cessation of 20-Hz click trains (red square in **A**) (blue for 7 fGluN1 controls; red for 6 mutants). Dotted lines: mean ± s.e.m. **(D)** The difference in the magnitude between 15 and 24 Hz power (arrowheads in **C**) from ISI spontaneous power for 20-Hz ASSRs was lower in mutant mice (red). ^*^p < 0.05, unpaired Student's *t*-test. **(E)** Phase locking to 20-Hz ASSR stimuli in control (blue) and mutant (red) mice. Dotted lines: mean ± s.e.m. **(F)** Magnitude of 15–24 Hz phase locking for 20-Hz ASSRs (arrowheads in **E**) was lower in mutant mice (red). ^*^p < 0.05 unpaired Student's *t*-test. Each dot represents individual animals. Dotted lines in **(C,E)** are s.e.m.

We next examined 20-Hz ASSRs (duration, 1000 ms) to explore whether ASSR deficits are specific to 40-Hz stimuli. Figure 2A depicts a representative example of the averaged evoked potentials (middle) and spectrogram (bottom) evoked by 20-Hz click trains (upper) in control (left) and mutant mice (right). First, we found no difference in the averaged N1 amplitudes between genotypes analyzed per animal (Figure 2B) or analyzed per channel (Supplemental Figure 2A). The difference in the evoked ASSR power, which were obtained by subtracting the spontaneous power amplitudes in between ASSR stimuli from the total ASSR power during last 200 ms before cessation of click-trains (dashed period in Figure 2A), also peaked at 20 Hz with a smaller peak at the 40 Hz harmonic in both genotypes (Figure 2C and Supplemental Figure 2B). However, the relative power of the dominant peak at 20 Hz (15–24 Hz) was lower in the mutants compared to controls per animal [Figure 2D, *t*_(11)_ = 2.59, *p* < 0.05, *d* = 1.47 (large effect size)] and in per-channel design [Supplemental Figure 2C, *t*_(55)_ = 4.58, *p* < 0.01, *d* = 1.25 (large effect size)]. Furthermore, phase locking of the 20-Hz ASSR consisted of a dominant peak at 20 Hz with several spectral peaks at harmonics of 20 Hz (Figure 2E and Supplemental Figure 2D). The dominant peak of phase locking factor at 15–24 Hz in the mutants was lower than the controls analyzed per animal [Figure 2F, *t*_(11)_ = 2.29, *p* < 0.05, *d* = 1.3 (large effect size)] and analyzed per channel [Supplemental Figure 2E, *t*_(55)_ = 3.77, *p* < 0.01, *d* = 1.01 (large effect size)], but other spectral peaks in the mutants were similar to those in controls per animal (*p* = 0.63 for 35–44 Hz, *p* = 0.37 for 55–64 Hz, *p* = 0.40 for 75–84 Hz, unpaired Student's *t*-test) and per channel (*p* = 0.44 for 35–44 Hz, *p* = 0.27 for 55–64 Hz, *p* = 0.50 for 75–84 Hz, unpaired Student's *t*-test). These results indicate both ASSR and phase-locking evoked by 20-Hz ASSR stimuli are also diminished in the mutant mice, while the magnitudes of auditory-evoked potentials triggered by 20-Hz stimuli are largely unaffected.

### Enhanced spontaneous LFP power in awake quiescent period

**Figure 3:**
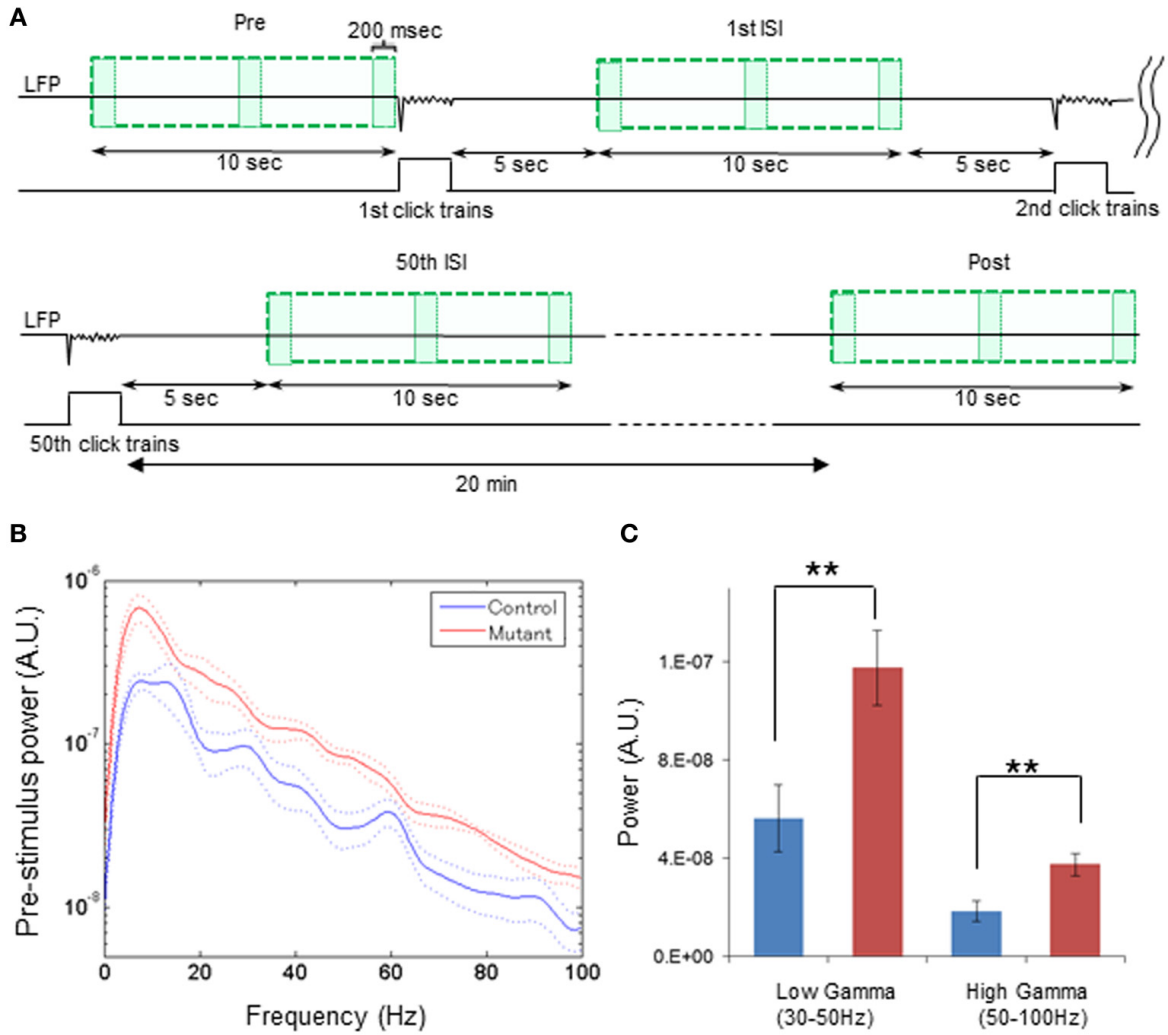
**Broadband elevation of mutant spontaneous LFP power during pre-stimulus period. (A)** Schematic diagram indicates the analysis periods of spontaneous LFP power during pre-stimulus period (Pre), following the first ASSR stimuli [1st inter-stimulus interval (ISI)], following the 50th ASSR stimuli (50th ISI), and during post-stimulus period (Post). For a pre-stimulus period, z-score normalized LFPs (top line) during last 10 s (upper left green square) before the first click train onset were analyzed with FFT algorism with every 200-ms bin (each box). During ASSR sessions, LFP data from 5 to 15 s (200-ms bin) after the 1st stimuli (upper right green square), the 25th stimuli (not shown), and 50th stimuli (bottom left green square) were analyzed with FFT algorithm. For a post-stimulus period, LFPs were obtained from a 10-s period (bottom right green square) 20 min after cessation of the last click trains were analyzed with FFT algorithm (200-ms bin). **(B)** Z-score normalized spectral density power during pre-stimulus period from control (blue) and mutant (red) mice (control: *n* = 7, mutant: *n* = 6). Dotted lines: mean ± s.e.m. A 60-Hz bump in control LFP power spectra was due to power line noise contamination. **(C)** Averaged spontaneous LFP powers at low gamma (30–50 Hz), high gamma (50–100 Hz) frequency range were higher in mutant (red) mice compared to control mice (blue). ^**^p < 0.01, unpaired Student's *t*-test.

To systematically explore the levels of spontaneous power throughout the periods of inter-ASSR stimulus intervals and the post-ASSR period, we further assessed the transition of spontaneous LFP power (z-score) from the pre-stimulus period to the inter-stimulus periods post to the first, 25th and 50th 40-Hz click-train administration, and the post-stimulus period 20 min after the cessation of last (50th) 40-Hz click-train (Figure 3A). First, we assessed the power spectra of z-score normalized LFPs during the pre-stimulus period from awake head-restrained animals. We found that baseline spontaneous power during the last 10-s pre-stimulus period prior to the first click-train administration was augmented in the mutants compared to the controls regardless of the spectral frequency found in both per-animal (Figure 3B) and per-channel (Supplemental Figure 3A) design. The intensities of averaged power for baseline LFPs at low gamma (30–50 Hz) and high gamma (50–100 Hz) range were both higher in the mutant mice compared to the controls per animal [Figure 3C, *t*_(11)_ = 3.00, *p* < 0.01, *d* = 1.67 for low gamma; *t*_(11)_ = 3.13, *p* < 0.01, *d* = 1.74 for high gamma] and per channel [Supplemental Figure 3B, *t*_(55)_ = 6.41, *p* < 0.01, *d* = 1.66 for low gamma; *t*_(55)_ = 5.56, *p* < 0.01, *d* = 1.46 for high gamma]. This elevation of LFP fluctuation continued even at super gamma frequency (100–120 Hz) per animal [*t*_(11)_ = 2.41, *p* < 0.05, *d* = 1.33 (large effect size)] and per channel [*t*_(55)_ = 4.68, *p* < 0.01, *d* = 1.24 (large effect size)], suggesting a broadband LFP power increase in the mutant animals during the awake quiescent period.

### Spontaneous LFP power returns back to normal upon ASSR stimuli

**Figure 4:**
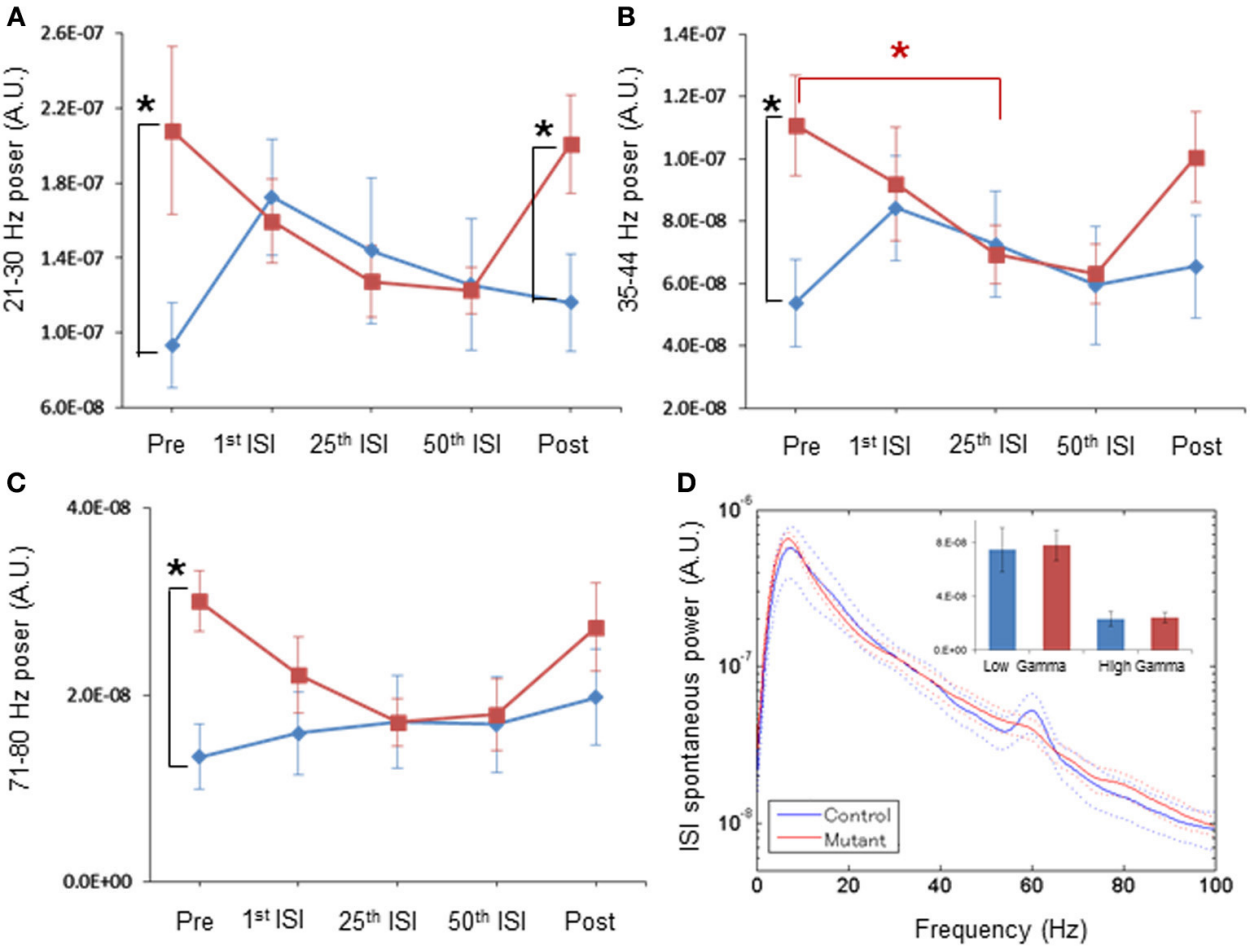
**Normal magnitude of baseline LFP power during periods of periodic ASSR stimuli, by per-animal design. (A)** Transition of z-score normalized spontaneous LFP powers per animal at 21–30 Hz frequency in control (blue) and mutant (red) mice during Pre (pre-stimulus period), 1st ISI (first inter-stimulus interval), 25th ISI, 50th ISI, and Post (post-stimulus period). ^*^p < 0.05. **(B)** Transition of spontaneous LFP powers at 35–44 Hz in control (blue) and mutant (red) mice. ^*^p < 0.05. **(C)** Transition of spontaneous LFP powers at 71–80 Hz in control (blue) and mutant (red) mice. ^*^p < 0.05. Repeated-measures ANOVA followed by *post-hoc* Bonferroni testing. **(D)** No differences in averaged spontaneous LFP power amplitudes in the first, the 25th and the 50th ISIs, across frequencies between control (blue, *n* = 7) and mutant (red, *n* = 6) mice. The inset shows no difference in average LFP power amplitudes at low gamma (30–50 Hz) and high gamma (50–100 Hz) frequency. A 60-Hz bump in control LFP power spectra was due to power line noise contamination. Dotted lines: mean ± s.e.m.

**Figure 5:**
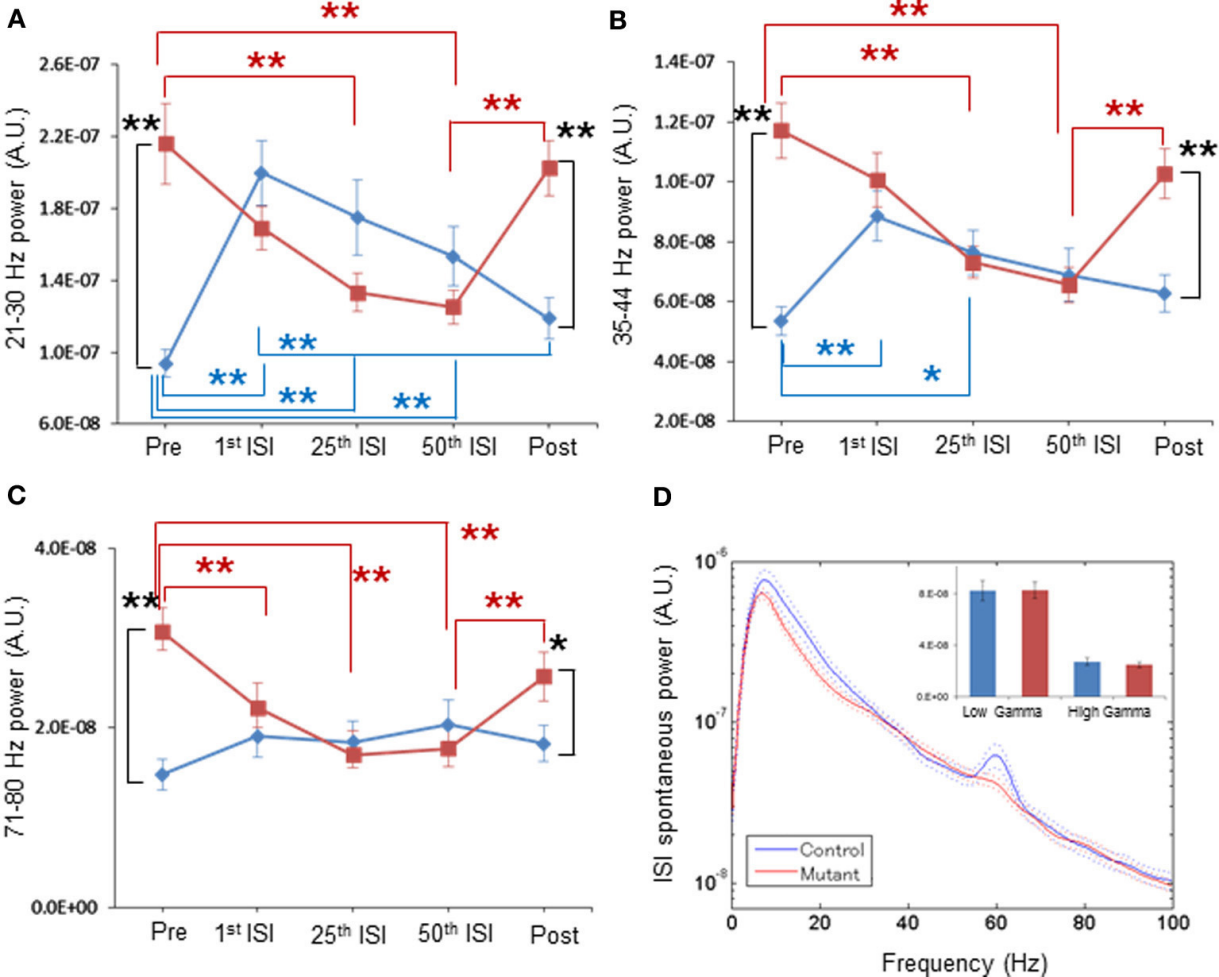
**Normal magnitude of baseline LFP power during periods of periodic ASSR stimuli, by per-channel design. (A)** Mean normalized powers in per channel design for 21–30 Hz frequency LFP fluctuation in control (blue, *n* = 31 sites from 7 animals) and mutant (red, *n* = 26 sites from 6 animals) mice during Pre-ASSR, 1st ISI, 25th ISI, 50th ISI, and post-ASSR. ^**^p < 0.01, repeated-measures ANOVA followed by *post-hoc* Bonferroni testing. **(B)** Mean normalized powers for 35–44 Hz frequency LFP fluctuation in control (blue) and mutant (red) mice during Pre-ASSR, 1st ISI, 25th ISI, 50th ISI, and Post-ASSR. ^**^p < 0.01 and ^*^p < 0.05, repeated-measures ANOVA followed by *post-hoc* Bonferroni testing. **(C)** Mean normalized powers for 71–80 Hz frequency LFP fluctuation in control (blue) and mutant (red) mice during Pre-ASSR, 1st ISI, 25th ISI, 50th ISI, and Post-ASSR. ^**^p < 0.01, ^*^p < 0.05, repeated-measures ANOVA followed by *post-hoc* Bonferroni testing. **(D)** No differences in averaged spontaneous LFP power amplitudes in the first, the 25th and the 50th ISIs, across frequencies between control mice (blue, *n* = 31 sites from 7 animals) and mutant mice (red, *n* = 26 sites from 6 animals). The inset shows no difference in average LFP power amplitudes at low gamma (30–50 Hz) and high gamma (50–100 Hz) frequency. A 60-Hz bump in control LFP power spectra was due to power line noise contamination. Dotted lines: mean ± s.e.m.

After the 40-Hz ASSR session began, we found a clear trend of a gradual reduction in mutant spontaneous LFP power amplitudes during inter-stimulus intervals with the increasing number of ASSR stimuli (Figures 4A–C, 5A–C). For example, spontaneous LFP power per animal was reduced in the inter-stimulus period following the 25th ASSRs, compared to the pre-ASSR period at 35–44 Hz [Figure 4B, *F*_(1, 11)_ = 1.389, *p* = 0.263 for genotype, Bonferroni *post-hoc* test, *p* < 0.05]. In per-channel design, spontaneous LFP power at 21–30 Hz [Figure 5A, *F*_(1, 54)_ = 1.326, *p* = 0.255 for genotype, Bonferroni *post-hoc* test, *p* < 0.05], 35–44 Hz [Figure 5B, *F*_(1, 54)_ = 6.392, *p* = 0.014 for genotype, Bonferroni *post-hoc* test, *p* < 0.05], and 71–80 Hz [Figure 5C, *F*_(1, 54)_ = 2.707, *p* = 0.106 for genotype, Bonferroni *post-hoc* test, *p* < 0.05], were all decreased by the 25th ISI in the mutants. On the other hand, the spontaneous LFP power in the control mice tended to increase after the 1st ASSR stimuli, particularly in beta frequency range (Figure 4A). This power increase upon ASSR stimuli in the control mice was prominent in the per-channel design (Figures 5A,B, *p* < 0.05, respectively). Consequently, no genotypic difference was detected in spontaneous LFP power magnitudes during 10-s inter-stimulus intervals (combined data of first, 25th and 50th ISIs) at any power spectra examined per animal (Figure 4D) and per channel (Figure 5D). Interestingly, 20-min after the last ASSR, the spontaneous LFP power in the mutants was significantly augmented [Figure 4A, *F*_(1, 11)_ = 1.104, *p* = 0.335 for genotype, Bonferroni *post-hoc* test, *p* < 0.05] to the level of pre-stimulus period. The elevation of LFP power amplitudes was more prominent in per channel analysis (Figures 5A–C, *p* < 0.05). These results suggest a brain state-dependent abnormality of baseline spontaneous LFP power in the mutant mice, i.e., an abnormally high spontaneous LFP power in an awake quiescent period, which disappears upon receiving external auditory stimuli. Our findings also strongly suggests that the evoked ASSR deficit found in our mutants is not due to greater baseline spontaneous gamma power, rather it is simply caused by the deficits in evoking responses by external stimuli.

## Discussion

We demonstrated abnormal oscillatory LFP power and impaired auditory-evoked LFP responses from the auditory cortex of awake, head-restrained GABA neuron-specific NMDAR hypofunction mice. Specifically, we found (1) a profound reduction of ASSR power and phase locking at 40-Hz, and to lesser degree, at 20-Hz, and (2) a broadband increase in spontaneous LFP power during the pre-stimulus period, but not during the inter-ASSR stimulus intervals. Interestingly, abnormal elevation of baseline spontaneous LFP power during the pre-stimulus period disappeared after the ASSR stimuli were presented. These finding suggest that NMDAR hypofunction in cortical GABAergic interneurons leads to two temporally distinct, brain state-dependent LFP deficits in A1 cortex; (1) the evoked ASSR deficits with normal level of spontaneous LFP power and (2) abnormal broadband elevation of spontaneous LFP power when no auditory stimuli are presented. Our study also showed no obvious contribution to the evoked ASSR deficits of augmented spontaneous LFP fluctuation following NMDAR hypofunction in GABAergic interneurons.

### Potential mechanisms underlying evoked ASSR deficits

We demonstrated robust ASSR deficits in the mutant mice in which NMDARs were selectively eliminated from 75 to 84% of PV-containing interneurons in neocortex (Belforte et al., 2010). This suggests that NMDARs in the PV-positive fast-spiking neurons are crucial for emergence of ASSRs. Optogenetically evoked-gamma oscillations have also been shown to be defective in mice in which NMDARs are genetically ablated from all PV-positive neurons (Carlén et al., 2012). The mechanism by which NMDAR deletion from PV neurons results in the ASSR deficits in not fully known. However, activation of cortical PV-positive interneurons in the thalamorecipient circuit is known to enhance acoustic information flow by feed-forward inhibition, which contributes to improved signal-to-noise ratio (Hamilton et al., 2013). In particular, the firing rate of fast-spiking neurons, likely PV-positive, appears to increase with increasing attention to external stimuli (Mitchell et al., 2007; Chen et al., 2008). It is noted that although selective genetic GluN1 deletion also occurs in ~30% of Reelin-positive interneurons in the mutant cortex, Reelin-positive neurons are located mostly in the supra-granular layers. Therefore, the most likely mechanism for our observation is a functional deficit in the NMDAR-deleted PV neurons that receive thalamocortical afferents. Presumed impairment in their feed-forward inhibition in response to acoustic stimuli may attenuate the generation of auditory evoked potentials followed by gamma oscillations. Further research exploring whether NMDAR hypofunction in cortical PV-neurons disturbs feedforward information flow elicited by auditory stimuli in A1 cortex is warranted.

### Potential mechanisms underlying the enhanced baseline LFP fluctuation

We also observed elevated spontaneous LFP oscillatory power in the pre-stimulus period before the animal attends to the auditory stimuli. Since genetic ablation of NMDARs selectively from PV neurons in awake mice also results in increased baseline power (Korotkova et al., 2010; Carlén et al., 2012), this finding is most likely due to NMDAR hypofunction in PV neurons. Similar results were also obtained from GluN1 hypomorph mice (Dzirasa et al., 2009; Gandal et al., 2012b) and from the acute administration of NMDAR antagonists (phencyclidine, ketamine, or MK-801) to rodents (Leung, 1985; Ma and Leung, 2000, 2007; Pinault, 2008; Ehrlichman et al., 2009; Hakami et al., 2009; Páleníček et al., 2011; Kulikova et al., 2012; Wood et al., 2012; Caixeta et al., 2013; Molina et al., 2014), to humans (Maksimow et al., 2006; Hong et al., 2010), and in *in vitro* slice preparation (McNally et al., 2011). The most likely mechanistic explanation for these effects is that cortical disinhibition elicited by NMDAR deletion from local PV neurons render the cortical glutamatergic neurons hyper-excitable (Olney and Farber, 1995; Homayoun and Moghaddam, 2007; Lisman et al., 2008; Nakazawa et al., 2012). However, the NMDAR hypofunction-induced baseline power increase is unlikely to be caused by hyper-synchrony of spiking activity. A recent *in vivo* unit/LFP recording study revealed that cortical disinhibition elicited by MK-801, a NMDAR antagonist, evoked an increase in the number of random spike trains of individual units and consequently a reduced synchronized firing of action potentials in mPFC of free-moving rats, despite a robust increase in LFP power at gamma frequency (Molina et al., 2014). This finding suggests a decoupling of gamma band LFP power from neuronal spiking synchrony. Similarly, we also previously reported in the same mutant mice used in this study there was a disruption in *in vivo* spike synchrony among pyramidal neurons in somatosensory cortex (Belforte et al., 2010). Therefore, the spontaneous LFP power increase following cortical NMDAR hypofunction may simply reflect a robust increase in synaptic inputs with aberrant or “noisy” spike firing. It is also plausible that NMDAR antagonism on GABAergic neurons in the basal ganglia and/or thalamic reticular nucleus causes disinhibition of thalamocortical neurons, leading to massive stimulation of cortical neurons at gamma frequency (Llinás and Ribary, 1993; Santana et al., 2011). However, this is unlikely in our model because the genetic manipulation is largely confined to the cortex and hippocampus.

### Brain state-dependent elevation of spontaneous LFP power

Unexpectedly, we found that spontaneous LFP power amplitudes tends to decrease during the repeated ASSR stimuli; a phenomenon which was more robust in per-channel design (Figure 5). Accordingly, the broadband elevation of spontaneous LFP power in the pre-stimulus period (Figure 3B) disappeared during the inter-ASSR stimulus periods (Figure 4D). The structure of cortical spontaneous activity is known to vary with cortical state or behavioral state (Steriade et al., 2001; Harris and Thiele, 2011). During the slow-wave sleep period and awake quiescent period, auditory cortex exhibits fluctuations of global activity between “synchronized” states of larger low frequency waves known as up and down state (Steriade et al., 1993; Harris and Thiele, 2011). In active wakefulness during tone presentation, these fluctuations are replaced by the “desynchronized” state characterized by low amplitude, high frequency LFPs (Castro-Alamancos, 2004). It has been reported that superficial pyramidal cells and putative fast-spiking neurons in rat A1 cortex dominate in awake quiescent period, and their activity was largely suppressed during auditory stimuli-induced cortical desynchronization (Sakata and Harris, 2012). The firing of fast-spiking neurons in rat somatosensory cortex, which is highly active during quiet wakefulness, is also dramatically suppressed during active whisking behavior (Gentet et al., 2010). Considering that the majority of cell-types in which NMDAR elimination occurred in our mutant mice are PV-positive fast-spiking neurons, it is conceivable that the state-dependent elevation of spontaneous LFP power reflects the dysfunction of mutant A1 cortex fast-spiking neurons during awake quiescent period. However, a recent study showed a dramatic increase in the putative fast-spiking neurons in visual cortex by the active running in a head-restrained condition that may elicit desynchronized state (Niell and Stryker, 2010). Further study is necessary to clarify the mechanisms of the state-dependent elevation of spontaneous LFP power observed in our mutant mice.

### Comparison to clinical data

Overall, the present results were consistent with the clinical EEG data showing reductions in the onset of auditory evoked responses (P50, N100) and of 40-Hz ASSR power and phase-locking in the cortex of individuals with schizophrenia, supporting the face validity of our mouse model. Furthermore, our findings argue against the possibility that 40-Hz ASSR deficits in patients with schizophrenia may reflect antipsychotic effects (Woo et al., 2010). However, we also found several findings inconsistent with the human data. First, in human adult subjects 40-Hz click trains induce the maximal ASSR at 40 Hz and the effects of 40-Hz stimuli at 20 and 30 Hz are smaller compared to 40 Hz (Galambos et al., 1981; Pastor et al., 2002; Picton et al., 2003). Since the optimal input frequency of fast-spiking neurons for action potential generation is known to be 30–50 Hz in rats (Pike et al., 2000), the ASSR impairment selectively at 40 Hz stimulation may suggest unequivocal deficits of fast-spiking neurons in patients with schizophrenia. In our study, however, 40-Hz stimuli induced a resonance peak at 30 Hz in addition to the 40-Hz peak (Figure 1D) whereas the phase locking spectrum showed a peak only at 40 Hz (Figure 1G). This may suggest that the murine A1 cortex exhibits a broader resonance frequency (30 Hz as well as 40 Hz) than in humans; although no power peak at 30 Hz was detected when stimulated at 20 Hz (Figure 2B). Further study is warranted to determine whether the resonant frequency to auditory stimuli is varied depending on the species.

Second, 20-Hz ASSRs are usually unaffected in schizophrenia (Kwon et al., 1999; Light et al., 2006; Vierling-Claassen et al., 2008); however some human ASSR studies also showed attenuation in 20-Hz ASSRs (Krishnan et al., 2009). In contrast, in our model 20 Hz ASSRs are reduced in power and phase locking (Figure 2). Nonetheless, the mutant ASSR peak at 20 Hz was still visible (Figure 2B) and attenuation of phase locking at 20 Hz was modest (Figure 2E), compared to robustness of 40-Hz ASSR deficits. Furthermore, the initial N1 responses triggered by 20-Hz ASSR stimuli were normal in the mutant mice. Therefore, the degree of evoked ASSR deficits appears to be more robust at 40-Hz than at 20-Hz in our mutant mice. It is conceivable that LFP recording directly from A1 cortex is more sensitive to detect ASSR impairment, compared to clinical skull-EEG recording.

Third, broadband enhancement of baseline EEG may not be characteristic of studies of resting EEG in patients with schizophrenia (Winterer et al., 2004; Kikuchi et al., 2011; Silverstein et al., 2012). However, Spencer (2012) re-analyzed their previous data which showed deficits in auditory evoked gamma oscillations and found that the pre-stimulus baseline gamma power was increased in the left auditory cortex of chronic patients. Interestingly, in his study, the baseline power increased across a wide frequency band (15–100 Hz) and this broadband increase was marginally significant, which is consistent with our finding.

Finally, this study involved relatively small sample sizes under per-animal analysis design (7 control and 6 mutant mice), which could be a confounding factor. However, nearly the same results were obtained by per-channel analysis (for example, Figure 4 vs. Figure 5), which further supports our conclusion.

### Clinical manifestation of baseline LFP power increase

Given that sensory-evoked gamma oscillation deficits are presumably linked to the cognitive deficits (Spencer et al., 2004; Cho et al., 2006), there are several possible clinical manifestation of baseline power increase. Increased baseline gamma oscillations have been reported in patients during psychotic episodes, including visual and auditory hallucinations (Baldeweg et al., 1998; Ropohl et al., 2004; Lee et al., 2006; Becker et al., 2009). Other studies suggest a link between baseline gamma oscillations and negative symptoms (Suazo et al., 2012), working memory (Winterer et al., 2004; Suazo et al., 2012), or synaptic plasticity (Bikbaev et al., 2008; Kulikova et al., 2012). A recent meta-analysis of functional neuroimaging in schizophrenia patients with auditory hallucinations revealed “paradoxical” engagement of A1 cortex, such that left A1 cortex displayed increased activation in the absence of external auditory stimuli (but with auditory verbal hallucinations), and decreased activation when an external stimulus was actually present (Kompus et al., 2011). Consistent with this, our mutant mice also exhibited an increase in baseline spontaneous LFP increase in the absence of external stimuli, which tended to decrease during repetitive ASSR stimuli and to return back to the elevated level 20 min after the last ASSR stimuli. This remarkable similarity between human patient studies and the finding in the present study may suggest that baseline LFP power increase is a signature of “paradoxical” A1 cortex activation in the absence of external stimuli. Further studies are warranted to assess the clinical and neurobiological significance of oscillations and synchrony deficits in schizophrenia.

## Author contributions

Kazuhito Nakao conceived and designed the study, performed the experiments, assembled, analyzed and interpreted the data, and wrote manuscript. Kazu Nakazawa conceived and designed the study, interpreted the data, and wrote the manuscript.

### Conflict of interest statement

The authors declare that the research was conducted in the absence of any commercial or financial relationships that could be construed as a potential conflict of interest.
