# Supplementary material for: Brain state-dependent abnormal LFP activity in the auditory cortex of a schizophrenia mouse model
Source: Front Neurosci. 2014 Jul 1;8:168. doi: 10.3389/fnins.2014.00168 (PMC4077015; doi:10.3389/fnins.2014.00168)
Supplement: Supplementary file 1 [file Presentation1.ZIP › Supp Mat legends.docx]

**Supplemental Figure 1**. **Both power and phase-locking of 40-Hz ASSRs are diminished in mutant mice**

(**A**) The averaged N1 amplitudes (z-score) per recording channel evoked by 40-Hz click were lower in the mutants compared to the control mice. Blue for 31 channels from 7 floxed-controls; red for 26 channels from 6 mutants. *p<0.05, unpaired Student’s *t*-test. (**B**) Evoked ASSR power (z-score) at 35-44 Hz frequency range during 40-Hz click train stimulation in per channel design in mutants (red) was lower than controls (blue). ***p*< 0.01, unpaired Student’s *t*-test. (**C**) The mean difference (A.U.) from ISI spontaneous power (green squire in Figure 1E) in click train-evoked ASSR power during last 200 ms before cessation of 40-Hz click trains (red square in Figure 1A and 1E) per recording channel. Dotted lines: mean ± SEM. (**D**) Magnitude difference of 35-44 Hz spectral power (arrowheads in Supplemental Figure 1C) from the ISI spontaneous power for 40-Hz ASSRs in mutants (red) was lower than controls (blue). ***p*< 0.01, unpaired Student’s *t*-test. (**E**) Phase locking to 40-Hz steady-state tone stimuli in control (blue) and mutant (red) mice. Dotted lines: mean ± SEM. (**F**) Magnitude of 35-44 Hz phase locking for 40-Hz ASSRs (arrowheads in Supplemental Figure E) in mutants (red) was lower than controls (blue). ***p*< 0.01, unpaired Student’s *t*-test. Each dot represents individual channels.

**Supplemental Figure 2**. **Both power and phase-locking of 20-Hz ASSR are diminished in mutant mice**

(**A**) No difference in the averaged N1 amplitudes (z-score) evoked by 20-Hz click trains between genotypes in per-channel design analysis (blue for 31 channels from 7 floxed-controls; red for 26 channels from 6 mutants). *p* = 0.38, unpaired Student’s *t*-test. (**B**) The mean difference (A.U.) from ISI spontaneous power in tone-evoked spectral power during last 200 ms before cessation of 20-Hz click trains (a red square in A) across all recording channels (blue for 31 channels from 7 floxed-controls; red for 26 channels from 6 mutants). Dotted lines: mean ± SEM. (**C**) Magnitude difference of 15-24 Hz power (arrowheads in Supplemental Figure 2A) from the ISI spontaneous for 20-Hz ASSRs in mutants (red) was lower than controls (blue). ***p*< 0.01, unpaired Student’s *t*-test. (**D**) Phase locking to 20-Hz steady-state tone stimuli in control (blue) and mutant (red) mice. Dotted lines: mean ± SEM. (**E**) Magnitude of 15-24 Hz phase locking for 20-Hz ASSRs (arrowheads in Supplemental Figure 2C) in mutants (red) was lower than controls (blue). ***p*< 0.01, unpaired Student’s *t*-test. Each dot represents individual channels.

**Supplemental Figure 3**. **Broadband elevation of mutant spontaneous LFP power during pre-stimulus period**

(**A**) Z-score normalized spectral density power during pre-stimulus period from control (blue) and mutant (red) mice (control: n=31 sites from 7 animals, mutant: n=26 sites from 6 animals). Dotted lines: mean ± SEM. (**B**) Mean normalized powers for low gamma (30-50 Hz) and high gamma (50-100 Hz) frequency LFP fluctuation were both higher in the mutant mice (red) compared to controls (blue). ***p*< 0.01, unpaired Student’s *t*-test.
