# Supplementary figures and images for: Brain state-dependent abnormal LFP activity in the auditory cortex of a schizophrenia mouse model
Source: Front Neurosci. 2014 Jul 1;8:168. doi: 10.3389/fnins.2014.00168 (PMC4077015; doi:10.3389/fnins.2014.00168)

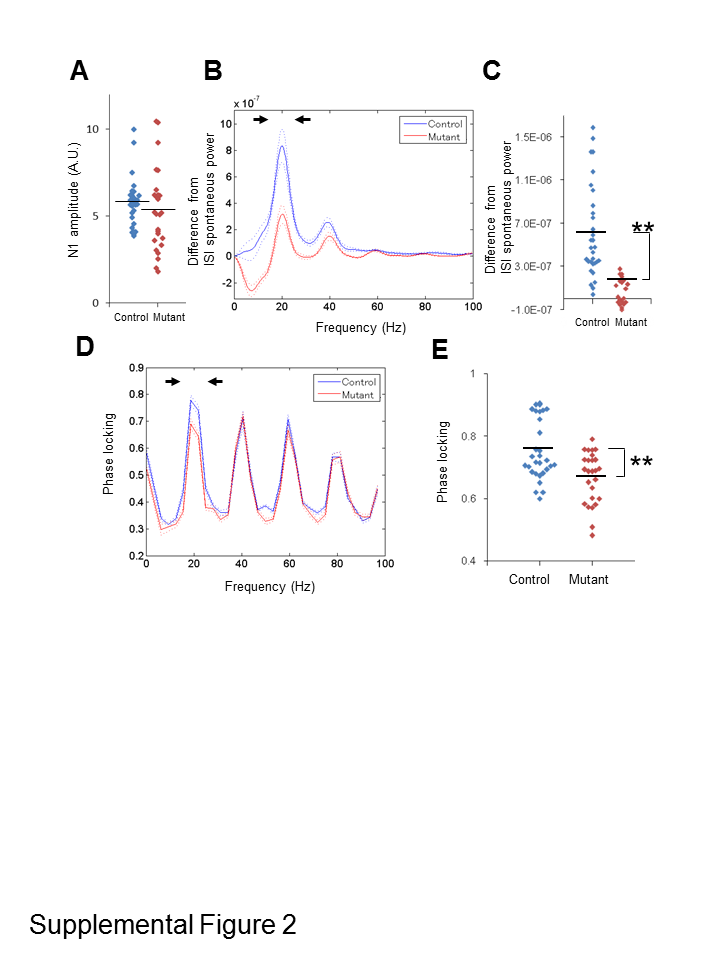

Supplement: Supplementary file 1 [file Presentation1.ZIP › Supp Fig 2.TIF]

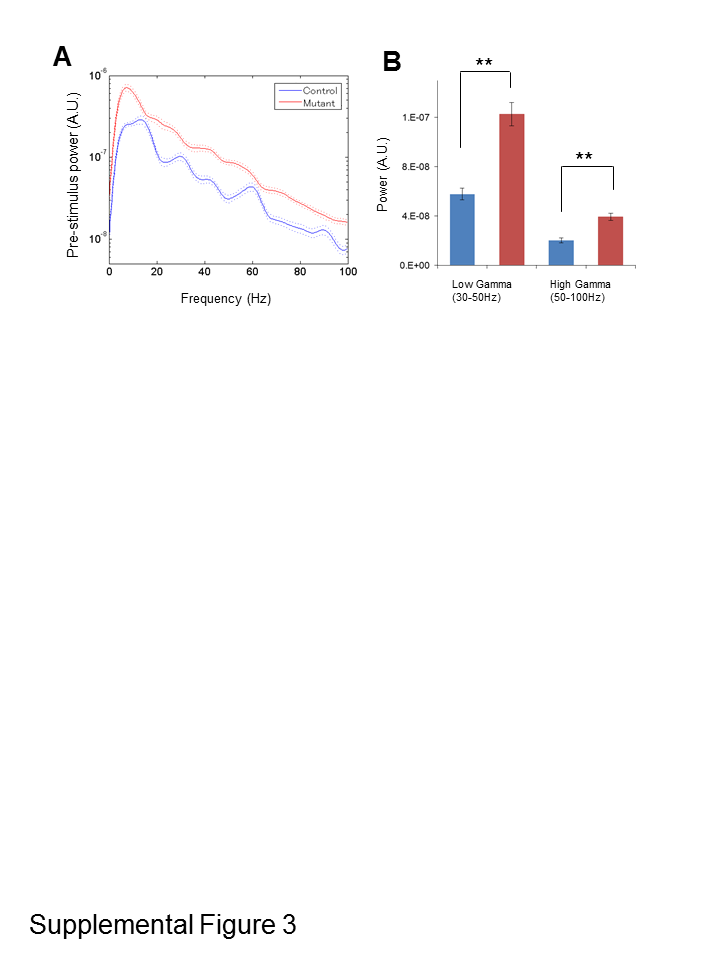

Supplement: Supplementary file 1 [file Presentation1.ZIP › Supp Fig 3.TIF]

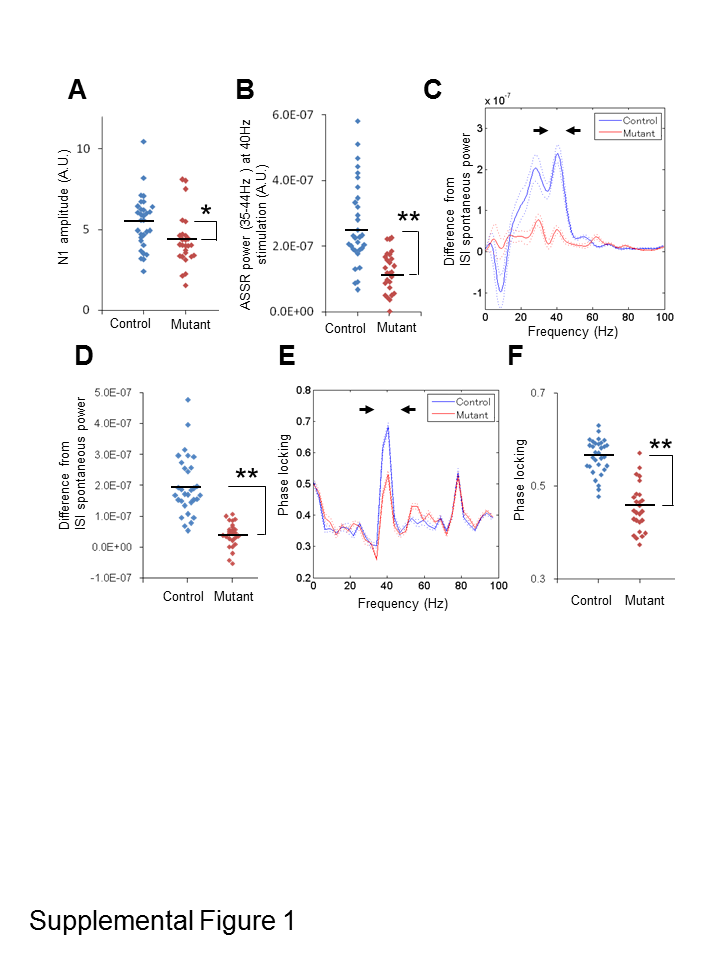

Supplement: Supplementary file 1 [file Presentation1.ZIP › Supp Fig 1.TIF]
